# In Silico Approach for Fluorene Biodegradation, and the Impacts of Derivatives on the Environment and Health

**DOI:** 10.3390/jox16020070

**Published:** 2026-04-20

**Authors:** Syed Raju Ali, Yasir Anwar, Hani Mohammed Ali

**Affiliations:** Department of Biological Sciences, Faculty of Science, King Abdulaziz University, Jeddah 21589, Saudi Arabia

**Keywords:** fluorene, biodegradation, toxicity, enzymes, metabolites, molecular docking

## Abstract

Fluorene poses ecological and health hazards that originate from biomass combustion and petroleum. However, some microorganisms can counter fluorene through complex enzymatic degradation pathways. This research aimed to explore the catalytic efficiency of enzymes on metabolites and their toxicity levels throughout the fluorene biodegradation pathway. Several web servers and software were used to characterize them and analyse molecular dockings between ligands and proteins. Fluorene and its metabolites have mild toxicities to the brain, lung, neurons, and kidneys, and consequent endpoints cause mutations, cancer, and ecotoxicity at different levels. The catalytic enzymes are well-folded, single-chained, medium-sized proteins that are acidic, thermostable, and with few exceptions, hydrophilic, cytoplasmic, non-allergenic, and nonvirulent, possessing multiple active sites. The ERRAT, PROCHECK, and VERIFY 3D tools successfully validated the SWISS-modelled 3D structures of proteins. Molecular docking results showed moderate binding affinities between proteins and ligands, ranging from −9.4 to −6.1 kcal/mol, indicating potential activities of the enzymes. This computational study supports the conventional fluorene degradation pathway and may provide a new avenue for further research.

## 1. Introduction

Polycyclic aromatic hydrocarbons (PAHs) are organic compounds composed of multiple fused benzene rings found in diverse ecosystems [1]. They are considered persistent environmental pollutants because of their toxic properties [2]. Fluorene is a low-molecular-weight PAH composed of two benzene rings connected by a carbon–carbon bond and an adjacent methylene bridge, which keeps it planar [3]. It is a hydrophobic white crystal, soluble in several organic solvents, and emits fluorescence [4,5].

Fluorene has multiple industrial applications, especially in dyes, pigments, agrochemicals, and pharmaceuticals, which enter the human body through skin absorption, inhalation, and oral administration [6]. The main sources of fluorene and other PAHs are incompletely combusted fossil fuels, plant biomass, and organic compounds [7]. PAHs are found broadly in industrial, urban, and coastal areas, binding with environmental pollutants, which makes them more rigid and toxic than before. The concentration of PAHs in Riyadh City, Saudi Arabia, measured in road dust between 0.01 and 126.0 ng/g (12.38–46.51 ng/g fluorene), and in air from 1.8 to 13.5 μg/m^3^ [8,9]. In the serum of asthmatic children in Saudi Arabia, fluorene was identified from 2.5 to 3.6 ng/mL [10]. In the USA, fluorene and derivatives were diagnosed in the urine of patients who had skin disease and obesity from 68.7 ng/L to 236.8 ng/L [11].

Fluorene is one of the 16 PAHs priority pollutants listed by the US Environmental Protection Agency, and it has harmful impacts on humans and the environment [12]. Several studies find that light PAHs, including fluorene, are more neurotoxic than heavier (four or more rings) PAHs [13]. The presence of fluorene and its derivatives was observed in the mouse brain and urine after inhalation and ingestion, which suggests toxicity for the human brain and a link to the marker of Alzheimer’s disease [14]. Higher fluorene exposure affects the human respiratory system and is associated with hypomethylation of F2RL3 and AHRR, which are marker genes that indicate lung cancer risk [15]. It is also associated with an extreme threat to kidney dysfunction and renal cancer [16]. Some fluorene derivatives are DNA intercalating agents that exhibit mutagenicity and carcinogenicity [17]. Moreover, fluorene induces oxidative stress, DNA damage, lipid peroxidation, protein carbonylation, and some enzymatic antioxidative activities in the earthworm *Eisenia fetida* and the marine worm *Perinereis aibuhitensis*, which are ecological biomarkers of fluorene ecotoxicity [18,19].

Remediation of PAHs, including fluorene, is challenging; nevertheless, biodegradation plays a vital role in eradicating toxic PAHs from the environment. Numerous PAH-degrading microorganisms have been isolated and characterized over the past few decades. A significant number of bacteria belong to the *Mycobacteriaceae*, *Nocardiaceae*, *Pseudomonadaceae*, and *Sphingomonadaceae* families [20]. Several fluorene-degrading bacterial species have been isolated, including *Pseudomonas frederiksbergensis* [21], *Nocardioides aquiterrae* [22], *Paenibacillus* sp. [23], *Gordonia polyisoprenivorans* [24], and *Terrabacter* sp. [25]. Furthermore, microfungi of the *Cunninghamella* genus, *Phanerochaete chrysosporium*, *Armillaria* sp., and *Mucor irregularis* also degrade fluorene [26,27,28,29].

Fluorene biodegradation involves multiple enzymatic catabolic reactions, and researchers have proposed several pathways over the years. Degradation is initiated through the oxygenation of fluorene by the enzyme DbfA into 9-fluorenol, which is dehydrogenated by the enzyme FlnB to 9-fluorenone. Subsequently, angular dioxygenation of 9-fluorenone is converted by the enzyme DbfA into 1,1a-Dihydroxy-1-hydrofluoren-9-one (DHF), which is again dehydrogenated by the enzyme FlnB to produce 2,3-Dihydroxy-2′-carboxybiphenyl (CDB). Then CBD is dioxygenated by the enzyme FlnD to 2-Hydroxy-6-oxo-6-(2-carboxyphenyl)-hexa-2,4-dienoate. Finally, the metabolite is hydrolysed by the enzyme FlnE to yield phthalate, which leads to benzoate degradation pathways. The corresponding enzymes in this pathway are: DbfA, fluorene 9-monooxygenase and 9-fluorenone 1,1a-dioxygenase; FlnB, DHF dehydrogenase; FlnD, CDB 1,2-dioxygenase; and FlnE, 2-hydroxy-6-oxo-6-(2′-carboxyphenyl)-hexa-2,4-dienoate hydrolase [30,31,32,33].

Fluorene biodegradation pathways have been broadly studied through traditional research, and numerous fluorene-degrading microorganisms and responsive enzymes have been identified. Nonetheless, significant gaps remain: to identify, for instance, (a) enzyme characterization at the molecular level, (b) successive fluorene biodegradation at each step, and (c) toxicological profiling of fluorene and intermediate metabolites. This study meets the remaining gaps through an inclusive in silico approach. The study addresses enzyme characterization, protein modelling, molecular docking at every step of the fluorene biodegradation pathway, and computational prediction of the toxicity of fluorene derivatives. An in silico study is cost-effective, time-saving, and supportive of conventional studies. However, computational analysis is not beyond limitations; to overcome them, it needs further study.

## 2. Methods and Materials

### 2.1. Retrieval of the Pathway Information

The fluorene biodegradation pathway was selected from the Kyoto Encyclopedia of Genes and Genomes (KEGG) (https://www.kegg.jp/entry/map00624 (accessed on 1 October 2025)). KEGG Pathway is a comprehensive bioinformatics database of metabolic maps, interactions, and networks of biomolecules and compounds [34]. From the database, fluorene and its subsequent intermediate derivatives, 9-Fluorenol, 9-Fluorenone, 1,1a-Dihydroxy-1-hydrofluoren-9-one, 2,3-Dihydroxy-2′-carboxybiphenyl, and 2-Hydroxy-6-oxo-6-(2-carboxyphenyl)-hexa-2,4-dienoate, along with the associated enzymes and genes, were retrieved. Based on information from the KEGG database, the fluorene degradation pathway is illustrated.

### 2.2. Substrates (Ligands) Extraction and Preparation

A total of six substrates related to the fluorene biodegradation pathway were used as ligands in molecular docking with proteins selected from the KEGG database. The molecular formulae, molecular weights, SMILES IDs, and 3D structures (SDF) of the substrates were retrieved from the PubChem database (https://pubchem.ncbi.nlm.nih.gov/ (accessed on 16 April 2026)). The PubChem database provides information and structures of numerous chemical compounds, maintained by NCBI [35]. The ligands were prepared at neutral physiological pH (7.0), energy was minimized using Swiss-PdbViewer (v4.1.0) software (http://www.expasy.org/spdbv/ (accessed on 5 October 2025)), and the data were converted to PDBQT format after geometry optimization.

### 2.3. Ligand Toxicity Determination

Fluorene is a polycyclic aromatic hydrocarbon that has toxic impacts on humans and the environment. The toxicity level of fluorene and its intermediate derivatives was determined using the web-based TOX-PREDICTION tool of the ProTox 3.0—Prediction of Toxicity of Chemicals server (https://tox.charite.de/protox3/index.php?site=compound_input (accessed on 20 October 2025)) [36]. The SMILES IDs were input into the tool, and the Organ Toxicity and Toxicity Endpoints parameters were selected. Notably, the ProTox 3.0 tool depends on machine learning and existing datasets. It generates an idea of toxicity that may not be like in vivo interactions.

### 2.4. Enzymes (Proteins) Sequence Retrieval

From the fluorene biodegradation pathway, a total of six enzymes were selected, and protein FASTA reference sequences with related information, including bacterial species and location of corresponding genes, were retrieved from the protein database of the National Center for Biotechnology Information (NCBI) (https://www.ncbi.nlm.nih.gov/ (accessed on 16 April 2026)) [37].

### 2.5. Physicochemical Properties Prediction of the Proteins

The physicochemical properties of the enzymes (proteins), including molecular formula, molecular weight, theoretical pI, instability index, aliphatic index, and GRAVY (grand average of hydropathicity), were calculated by submitting FASTA sequences to the Expasy ProtParam tool of the Swiss Bioinformatics Resource portal (https://web.expasy.org/protparam/ (accessed on 20 October 2025)), as it calculates the physical and chemical properties of protein [38].

### 2.6. Localization, Allergenicity, and Virulence Characterization

The locations of the proteins were identified using the web-based subcellular localisation system tool CELLO (https://cello.life.nctu.edu.tw/ (accessed on 21 October 2025)). FASTA sequences of the proteins were submitted, and the organisms (Gram-positive or Gram-negative) were selected for accurate prediction [39]. Additionally, the allergenicity and virulence activities of the chosen proteins were predicted using AllerTOP v2.1 (https://www.ddg-pharmfac.net/allertop_test (accessed on 22 October 2025)) [40] and VirulentPred 2.0 (https://bioinfo.icgeb.res.in/virulent2/ (accessed on 22 October 2025)) [41] servers, respectively.

### 2.7. Homology Modelling of Proteins and Active-Site Prediction

The three-dimensional (3D) structures of proteins were modelled by using the SWISS-MODEL tool of the SWISS-MODEL Repository (https://swissmodel.expasy.org/ (accessed on 16 April 2026)). FASTA reference sequences of proteins were inserted into the tool, and more identical and reliable templates were selected to build protein models. The template coverage, Global Model Quality Estimate (GMQE), sequence similarity (identity), and methods of structure prediction are shown in Table 1. The best 3D protein structures were saved in PDB format [42]. Meanwhile, the active sites of proteins were identified by a web-based interface tool called PrankWeb (https://prankweb.cz/ (accessed on 25 October 2025)). The PBD files of the modelled proteins were uploaded to the server as ‘Custom structure’ to predict the active binding site(s). The PrankWeb predicts the active binding sites of protein structures, using the P2Rank machine learning method [43].

### 2.8. Validation of Predicted 3D Structures of Proteins

The quality of 3D structures of modelled proteins was assessed using the ERRAT, PROCHECK, and VERIFY 3D tools of UCLA-DOE LAB—SAVES v6.1 (https://saves.mbi.ucla.edu/ (accessed on 16 April 2026)). The PDB formats of the proteins were uploaded to the server; ERRAT calculated the locations of amino acids in error or non-error regions and represented them in a graph, whereas PROCHECK evaluated the 3D structure based on the distribution of amino acid residues at different categories on the Ramachandran plot. The VERIFY 3D tool calculated the average 3D-1D score ≥ 0.1 (in percentage) of amino acid residues of proteins. All evaluations helped to find the accuracy of the targeted proteins [44].

### 2.9. Molecular Docking Between Ligands and Proteins, and Visualization

The ligands and proteins of the fluorene biodegradation pathway were selected for molecular docking analysis. The dockings were performed using a web-based server, PrankWeb (P2RANK) (https://prankweb.cz/ (accessed on 28 October 2025)). The PDB files of proteins were uploaded to the Custom structure tool, and the ligand SMILES IDs were inserted into the docking task site. Molecular docking between proteins and ligands was also performed through PyRx (0.8) software (https://pyrx.sourceforge.io/ (accessed on 30 October)) [45]. Proteins were converted to PDBQT files after energy minimization using Swiss-PdbViewer (v4.1.0). The proteins and ligands were input into PyRx, and proteins were converted to macromolecules. The maximum grid box extent was selected for each docking to facilitate the ligands into targeted active sites (Table 2). The exhaustiveness was set to 8 by default, and 9 binding modes generated per ligand were stored in CSV format [46]. The docking complex with the lowest energy (most negative) and the Root Mean Square Deviation lower-bound (RMSD/lb) and upper-bound (RMSD/ub) close to 0.0 Å were collected for each pair. The results from PrankWeb and PyRx were downloaded, and using PyMOL software (v2.5.5), the PDBQT files for each protein and ligand were combined into PDB files (https://pymol.org/ (accessed on 16 April 2026)) [47]. The PyMOL-mediated PDB files were opened in BIOVIA Discovery Studio Visualizer software (v20.1.0) (https://www.3ds.com/products/biovia/discovery-studio (accessed on 1 November 2025)) [48]. The 2D and 3D structures of amino acid residues with ligand binding were saved in image format.

## 3. Results

### 3.1. Data Retrieval from the Fluorene Biodegradation Pathway

**Figure 1:**
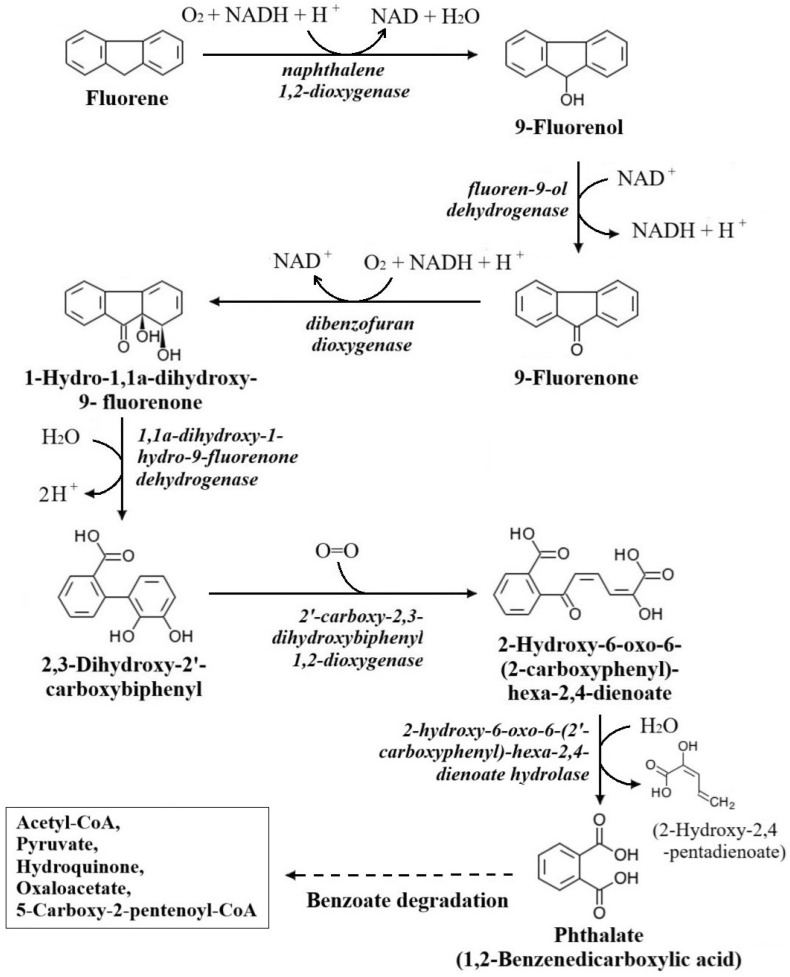
Fluorene biodegradation pathway according to the KEGG database. *The reaction arrows indicate the consecutive enzymatic degradation of fluorene into intermediates, the annotated (curve) arrows indicate the reaction cofactors (blunt ends) and the reaction byproducts (sharp ends), and the dashed arrow indicates the following pathways*.

There are three short and long defined pathways for fluorene biodegradation in the KEGG (map00624) database. The longest chain reaction pathway, excluding side branches, was selected for this study (Figure 1). From the metabolic pathway, six consecutive reactions along with related substates (Fluorene, 9-Fluorenol, 9-Fluorenone, 1,1a-Dihydroxy-1-hydrofluoren-9-one, 2-Hydroxy-6-oxo-6-(2-carboxyphenyl)-hexa-2,4-dienoate) and enzymes (naphthalene 1,2-dioxygenase, fluoren-9-ol dehydrogenase, dibenzofuran dioxygenase, 1,1a-dihydroxy-1-hydro-9-fluorenone dehydrogenase, 2′-carboxy-2,3-dihydroxybiphenyl 1,2-dioxygenase, 2-hydroxy-6-oxo-6-(2′-carboxyphenyl)-hexa-2,4-dienoate hydrolase) were targeted. At the end of this pathway was a single-ring phthalate, which further undergoes the benzoate degradation pathway.

### 3.2. Ligands (Substrates) Selection

The substrates were selected from the fluorene biodegradation pathway (KEGG #map00624) as ligands. The 3D structures (SDF) were downloaded, and SMILES IDs and molecular weights were collected, as shown in Table 3.

### 3.3. Toxicity of Ligands

The TOX-PREDICTION server tool was used to predict the probable toxicity of the ligands. The toxicity levels of ligands are in classes 4 and 5, with the lethal dose (LD) 50 ranging from 487 to 5000 mg/kg. They are toxic to organs, for example, neurons, the respiratory system, and the kidneys. The toxicity endpoints may cause cancer, mutations in genetic materials, penetration of the blood–brain barrier (BBB), and ecotoxicity, as shown in Table 4. There are several software and web tools available to determine the toxicity of chemicals and compounds, such as ProTox 3.0, Discovery Studio’s TOPKAT (v3.5), Toxicity Estimation Software Tools (T.E.S.T.) (v5.1.2), Tox21 dataset, and SuperToxic. Among them, ProTox 3.0 was chosen because it is easily accessible, free of cost, multidimensional, and sensitive and specific to respective models [36,49].

### 3.4. Protein Sequence Retrieval

The (protein) FASTA reference sequences with accession numbers of the selected enzymes were retrieved from NCBI. The number of amino acids of proteins, gene names, locations, source species, and taxonomic groups are shown in Table 5. The genes are located on plasmids (Naphthalene 1,2-dioxygenase and 2-hydroxy-6-oxo-6-(2′-carboxyphenyl)-hexa-2,4-dienoate hydrolase) and chromosomes of (most) Gram-positive and Gram-negative bacteria.

### 3.5. Physicochemical Properties of the Proteins

The Expasy ProtParam was used to predict the physicochemical properties of the proteins, as shown in Table 6. According to molecular weights, the proteins (30.71–49.77 kDa) are generally soluble in water. They are acidic in nature (theoretical pI: 4.69–5.89), which enhances solubility in the cytoplasm. The instability indices (28.84–38.25) are below the threshold (40), suggesting proteins are structurally stable. Aliphatic indices range between 72.79 and 93.28, indicating that all are thermostable under varying environmental conditions [50]. The negative GRAVY scores (−0.514 to −0.155) suggest that the proteins are hydrophilic, whereas 2′-carboxy-2,3-dihydroxybiphenyl 1,2-dioxygenase (GRAVY: 0.124) is mildly hydrophobic.

### 3.6. Protein Localization, Allergenicity, and Virulence Properties Prediction

According to the CELLO prediction, most proteins are cytoplasmic except for naphthalene 1,2-dioxygenase (extracellular). The AllerTOP predicted that most proteins are non-allergenic, but dibenzofuran dioxygenase is allergenic. The VirulentPred predicted that Fluoren-9-ol dehydrogenase and 2′-carboxy-2,3-dihydroxybiphenyl may be virulent, but other proteins are nonvirulent.

### 3.7. Homology Modelled Proteins and Predicted Active-Sites

**Figure 2:**
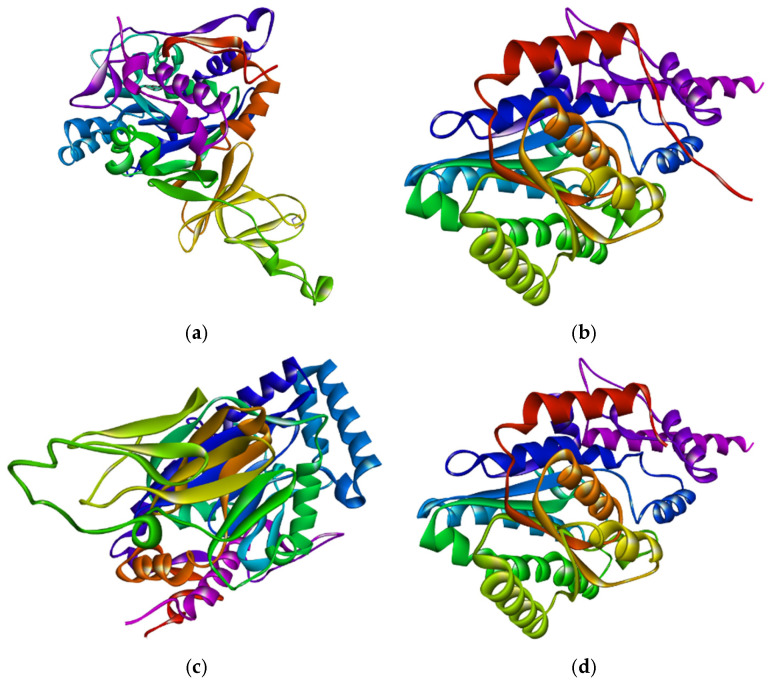

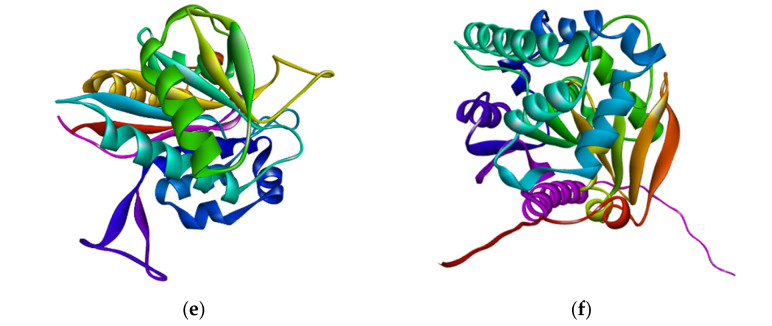
The 3D structures of SWISS-Model proteins. (**a**) Naphthalene 1,2-dioxygenase, (**b**) Fluoren-9-ol dehydrogenase, (**c**) Dibenzofuran dioxygenase, (**d**) 1,1a-dihydroxy-1-hydro-9-fluorenone dehydrogenase, (**e**) 2′-carboxy-2,3-dihydroxybiphenyl 1,2-dioxygenase, and (**f**) 2-hydroxy-6-oxo-6-(2′-carboxyphenyl)-hexa-2,4-dienoate hydrolase. *Different colors indicate individual peptide chains*.

**Figure 3:**
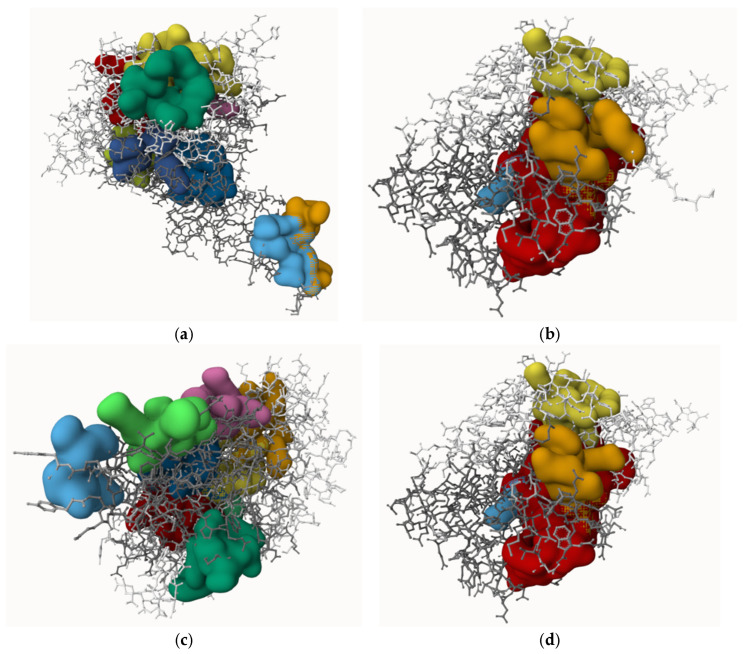

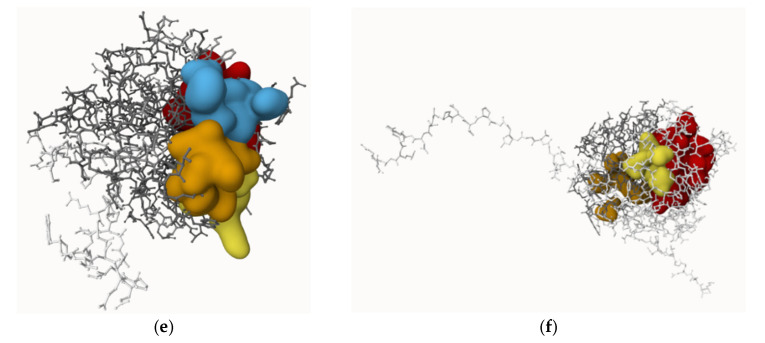
The active sites of the proteins. (**a**) Naphthalene 1,2-dioxygenase, (**b**) Fluoren-9-ol dehydrogenase, (**c**) Dibenzofuran dioxygenase, (**d**) 1,1a-dihydroxy-1-hydro-9-fluorenone dehydrogenase, (**e**) 2′-carboxy-2,3-dihydroxybiphenyl 1,2-dioxygenase, and (**f**) 2-hydroxy-6-oxo-6-(2′-carboxyphenyl)-hexa-2,4-dienoate hydrolase. *Different colors indicate individual active sites*.

The protein 3D structures were predicted using the SWISS-Model tool. The structures were generated and completed based on 100% coverage and 87.93–100.0% identical templates, as shown in Table 1. Each protein consists of a single chain, and the modelled 3D structures of all are well-folded, as shown in Figure 2. The active (binding) sites of the proteins were predicted by using the PrankWeb tool, shown in Figure 3. The proteins contain between three and nine active sites. The highest scores of the active sites, probability, number of residues, and average conservation are presented in Table 7. The scores of active sites provide insight into the functional regions of the proteins and indicate the potential efficiency for ligand binding.

### 3.8. The Features of Predicted 3D Structures of Proteins

**Figure 4:**
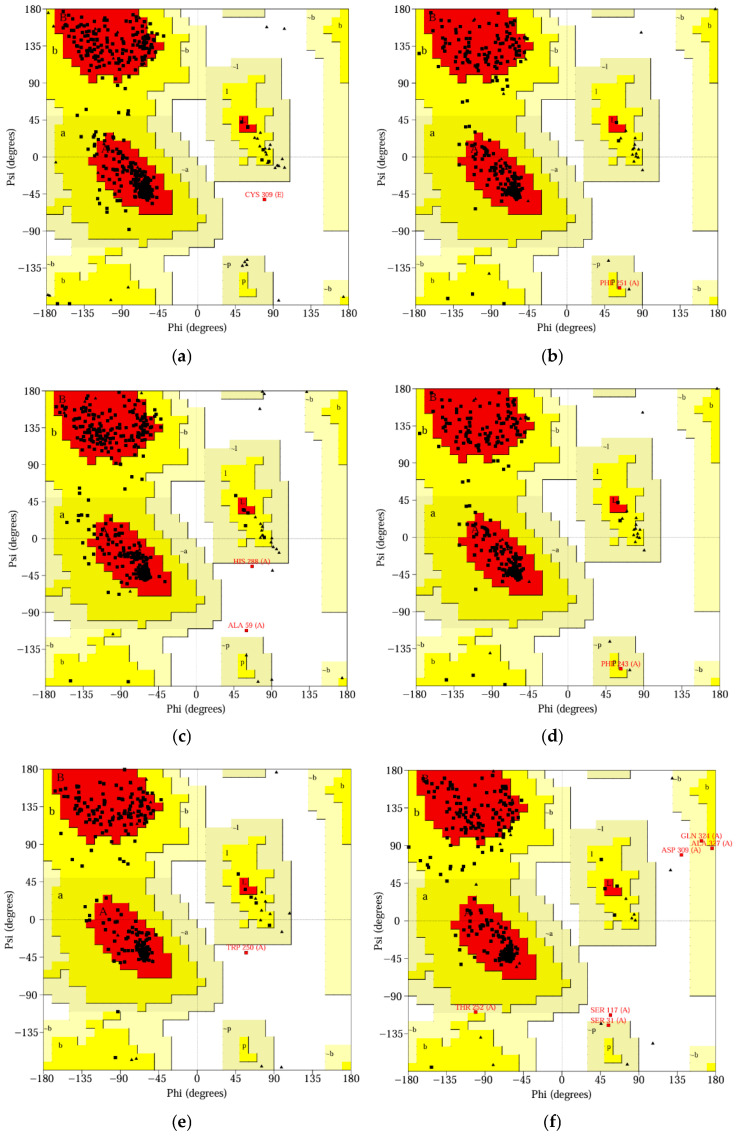
PROCHAKE-generated Ramachandran plots presenting the distribution of amino acid residues in different regions. (**a**) Naphthalene 1,2-dioxygenase, (**b**) Fluoren-9-ol dehydrogenase, (**c**) Dibenzofuran dioxygenase, (**d**) 1,1a-dihydroxy-1-hydro-9-fluorenone dehydrogenase, (**e**) 2′-carboxy-2,3-dihydroxybiphenyl 1,2-dioxygenase, and (**f**) 2-hydroxy-6-oxo-6-(2′-carboxyphenyl)-hexa-2,4-dienoate hydrolase. *[A,B,L] indicates the residues in most favoured regions (red color), [a,b,l,p] indicates the residues in additional allowed regions (deep yellow color), [~a,~b,~l,~p] indicates the residues in generously allowed regions (light yellow color), and white color indicates the residues in disallowed regions*.

Using the UCLA-DOE LAB-SAVES server, the 3D structures of the proteins were assessed. The ERRAT tool predicted proteins’ overall quality factors ranging from 94.279 to 99.403. On the other hand, the PROCHECK tool validated the proteins through the RAMACHANDRAN plots. The plots show that 86.2% to 93.3% of the amino acid residues of the proteins are in the most allowed regions, 6.3% to 11.6% in additional allowed regions, 0.0% to 1.4% in the generously allowed regions, and 0.0% to 1.4% in the disallowed areas (Figure 4). The VERIFY 3D tool predicted that 81.38% to 97.53% of amino acid residues have an average 3D-1D score ≥ 0.1, indicating that all proteins pass the lowest threshold (80%). The results of both predictions indicate the proper validation of the 3D protein structures, as shown in Table 8.

### 3.9. Molecular Docking Between Ligands and Proteins

The PrankWeb server tool and PyRx (AutoDock Vina) software (v0.8) were used to execute docking between proteins and ligands. Effectively, each ligand bound to the respective protein in both docking analysers (PrankWeb and PyRx), shown in Table 9. Fluorene showed the lowest docking energy with naphthalene 1,2-dioxygenase (−9.418 Kcal/mol in the PrankWeb and −9.4 Kcal/mol in PyRx). On the other hand, 2′-carboxy-2,3-dihydroxybiphenyl showed moderate binding affinity with 2,3-dihydroxy-2′-carboxybiphenyl in the PrankWeb tool (−6.452 Kcal/mol), and 2-Hydroxy-6-oxo-6-(2-carboxyphenyl)-hexa-2,4-dienoate showed the least binding affinity with 2-hydroxy-6-oxo-6-(2′-carboxyphenyl)-hexa-2,4-dienoate hydrolase in PyRx software (v0.8) (−6.1 kcal/mol) but more affinity in the PrankWeb tool (−7.351 Kcal/mol). The RMSD for the upper-bound and lower-bound was 0.0 Å at each docking, indicating top-ranked binding, stable, and reproducible outcomes without alternate conformations. It can be noticed that the PrankWeb tool was easier to access and saved time compared to the PyRx software (v0.8) during the docking study.

### 3.10. Binding Interaction Analysis

**Figure 5:**
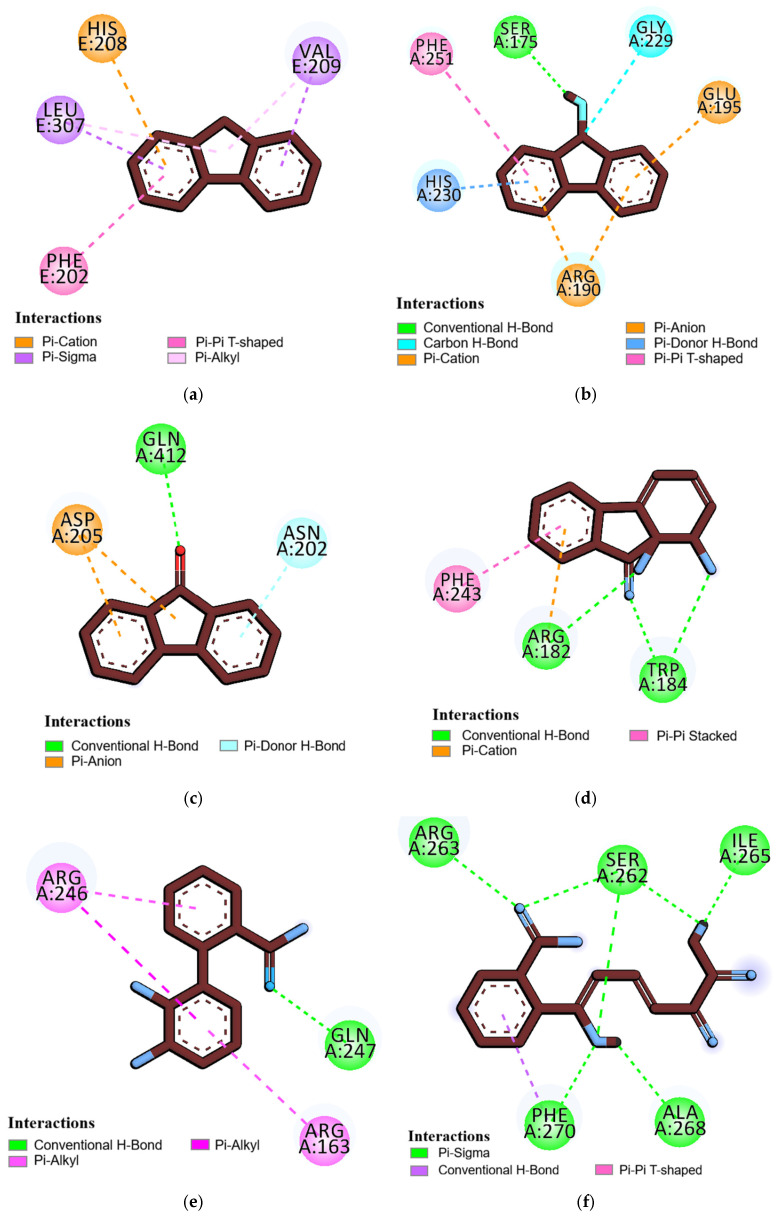
The 2D structures of the docked complex binding interactions between ligand and amino acid residues of proteins. (**a**) Fluorene with Naphthalene 1,2-dioxygenase, (**b**) 9-Fluorenol with Fluoren-9-ol dehydrogenase, (**c**) 9-Fluorenone with Dibenzofuran dioxygenase, (**d**) 1,1a-Dihydroxy-1-hydrofluoren-9-one with 1,1a-dihydroxy-1-hydro-9-fluorenone dehydrogenase, (**e**) 2,3-Dihydroxy-2′-carboxybiphenyl with 2′-carboxy-2,3-dihydroxybiphenyl 1,2-dioxygenase, and (**f**) 2-Hydroxy-6-oxo-6-(2-carboxyphenyl)-hexa-2,4-dienoate with 2-hydroxy-6-oxo-6-(2′-carboxyphenyl)-hexa-2,4-dienoate hydrolase. *Dash lines indicate the interactions between ligand and amino acid residues*.

**Figure 6:**
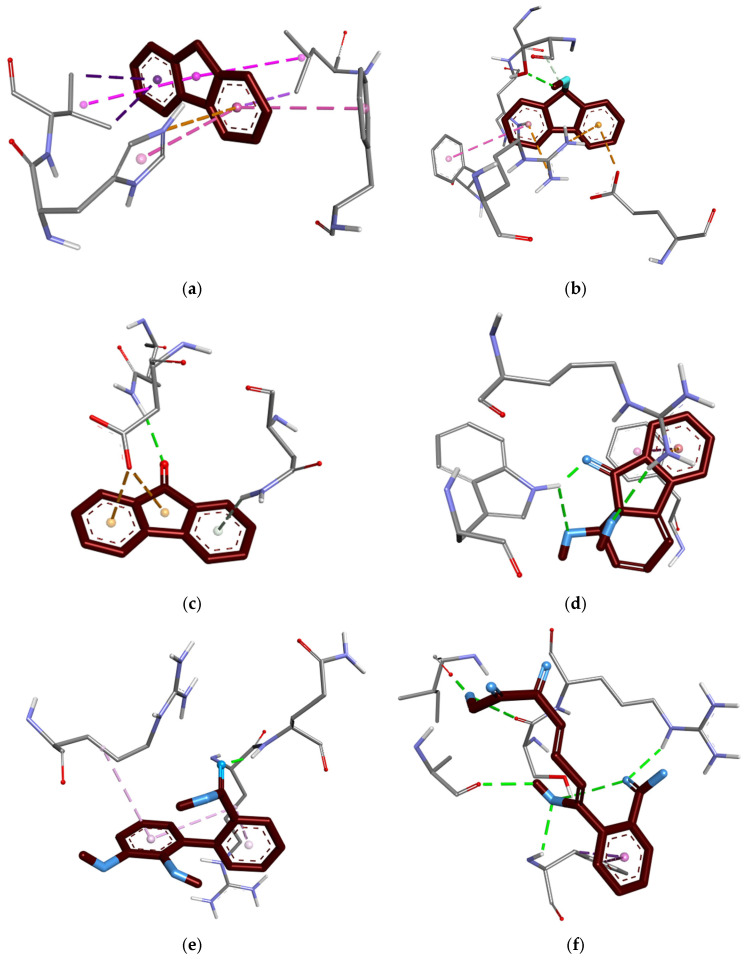
The 3D structures of the docked complex binding affinity between ligand and amino acid residues in PyRx software (v0.8). (**a**) Fluorene with Naphthalene 1,2-dioxygenase (−9.4), (**b**) 9-Fluorenol with Fluoren-9-ol dehydrogenase (−7.7), (**c**) 9-Fluorenone with Dibenzofuran dioxygenase (−6.6), (**d**) 1,1a-Dihydroxy-1-hydrofluoren-9-one with 1,1a-dihydroxy-1-hydro-9-fluorenone dehydrogenase (−8.5), (**e**) 2,3-Dihydroxy-2′-carboxybiphenyl with 2′-carboxy-2,3-dihydroxybiphenyl 1,2-dioxygenase (−6.2), and (**f**) 2-Hydroxy-6-oxo-6-(2-carboxyphenyl)-hexa-2,4-dienoate with 2-hydroxy-6-oxo-6-(2′-carboxyphenyl)-hexa-2,4-dienoate hydrolase (−6.1). *Dash lines indicate the interactions between ligand and amino acid residues*.

BIOVIA Discovery Studio software (v20.1.0) was used to find the binding interactions between ligands and amino acid residues of proteins. Fluorene possesses hydrophobic interactions through an electrostatic charge with PHE E:202 (4.76 Å), pi sigma bonds with LEU E:307 (3.63 Å) and VAL E:209 (4.83 Å), and a pi cation charge with HIS E:208 (4.86 Å), but no conventional hydrogen bond. Other ligands exhibit both classical and nonclassical hydrogen bonds, as well as hydrophobic (π) charge interactions and unfavourable donor–acceptor bonds with the amino acid residues of corresponding proteins, as shown in Table 10 and Figure 5 and Figure 6.

## 4. Discussion

Biodegradation of fluorene produces key metabolites, including 9-fluorenol, 9-fluorenone, 1,1a-Dihydroxy-1-hydrofluoren-9-one, 2,3-Dihydroxy-2′-carboxybiphenyl, and 2-Hydroxy-6-oxo-6-(2-carboxyphenyl)-hexa-2,4-dienoate [30,32]. The ring cleavage reactions gradually increase molecular weight; for example, fluorene weighs 166.22 g/mol, while 2-Hydroxy-6-oxo-6-(2-carboxyphenyl)-hexa-2,4-dienoate weighs 260.20 g/mol (Table 3).

Overall, fluorene and its metabolites cause neurotoxicity, blood–brain barrier penetration [13], respiratory toxicity [15], nephrotoxicity [16], mutagenicity, carcinogenicity [17], and ecotoxicity [18]. This research found that fluorene and its metabolites have class 4 and 5 toxicities at the lethal dose (LD50), ranging from 487 to 5000 mg/kg, indicating mild toxicity. Fluorene, 9-fluorenol, and 9-fluorenone cause neurotoxicity with a probable range of 0.52 to 0.76; 1,1a-Dihydroxy-1-hydrofluoren-9-one and 2,3-Dihydroxy-2′-carboxybiphenyl cause respiratory toxicity with a probability of 0.67 and 0.61, respectively; and 2,3-Dihydroxy-2′-carboxybiphenyl and 2-Hydroxy-6-oxo-6-(2-carboxyphenyl)-hexa-2,4-dienoate with a probability of 0.66 and 0.64, respectively. The endpoints of fluorene and most metabolites cause carcinogenicity (probability 0.53 to 0.81), mutagenicity (probability 0.50 to 0.79), blood–brain barrier penetration (probability 0.61 to 0.97), and ecotoxicity (probability 0.69 to 0.84). Generally, in laboratories, the concentration of fluorene derivatives and other PAHs is diagnosed using gas chromatography–mass spectrometry (GC-MS), and high-performance liquid chromatography (HPLC) at a 255 nm wavelength [29]. Though lethal doses of fluorene derivatives have been identified as 487 to 5000 mg/kg, only 0.32 to 0.37 mg/kg fluorene caused lung cancer risk to creosote-exposed workers, and 2.5–3.6 ng/mL fluorene caused childhood asthma [10,15]. On the other hand, green algae *Chlorella vulgaris* were sensitive to >1.0 mg/L, and female CD-1 mice had leukocytic and cytoplasmic disorders at 2.0 to 50 mg/kg fluorene derivatives [51,52].

Bacteria such as *Pseudomonas* sp. [21], *Nocardioides aquiterrae* [22], *Paenibacillus* sp. [23], *Gordonia polyisoprenivorans* [24], *Terrabacter* sp. [25], and *Vibrio cyclotrophicus* [53] degrade PAHs, including fluorene. They carry genes on plasmids and chromosomes, and most PAH-degrading enzymes are translated from individual genes. However, fluoren-9-ol dehydrogenase and 1,1a-dihydroxy-1-hydro-9-fluorenone dehydrogenase are from a single gene, *flnB*, with different amino acid numbers. Most enzymes are non-allergenic and nonvirulent, ensuring the health safety of PAH bioremediation from the environment [54].

The proteins are single-chained, medium in size (30.71–49.77 kDa), acidic (pI: 4.69–5.89), and mostly hydrophilic (GRAVY score: −0.514 to −0.155), with instability indexes (28.84–38.25, below the threshold 40) and aliphatic indexes (72.79–93.28) that indicate they are water soluble, thermostable and can function in diverse stressed conditions [55]. In the Protein Data Bank (PDB) database, the information and 3D structures of proteins are not available (except for naphthalene 1,2-dioxygenase). As a result, the SWISS-Model tool was used to retrieve the 3D structures. The templates covered 100% sequences and 87.93–100.0% similarity, indicating higher accuracy of modelled proteins, which were validated using the UCLA-DOE LAB-SAVES server. The ERRAT scores (94.279 to 99.403), PROCHECK scores (over 90%), and VERIFY 3D scores (81.38% to 97.53%) of amino acid residues confirm the high accuracy of 3D structures [56]. The proteins have multiple active binding sites (3 to 9), which allow them to bind with ligands.

Molecular docking between proteins and ligands was performed using the PrankWeb server and PyRx (v0.8) to ensure accurate results. Firstly, the docking results (−9.418 to −6.1 Kcal/mol) indicate efficient binding interactions between ligands and amino acid residues of the proteins. Secondly, docking results from the PrankWeb server and PyRx software (v0.8) were almost similar except for 2-Hydroxy-6-oxo-6-(2-carboxyphenyl)-hexa-2,4-dienoate (PrankWeb: −7.351, PyRx: −6.1). Dallakyan and Oldon (2015) observed that docking results between −10.0 and −6.0 kcal/mol had moderate protein–ligand (enzyme–substrate) interactions experimentally [45]. Molecular docking indicates potential binding affinities between enzymes and fluorene derivatives; however, it does not confirm enzymatic catalytic activities [57]. The binding between ligands and amino acid residues occurs through hydrogen bonds, hydrophobic interactions, and electrostatic charges. Fluorene is hydrophobic [4], so it interacts with amino acid residues through hydrophobic bonds and electrostatic (π) charge, but not through hydrogen bonds. Nonetheless, other metabolites interact with amino acid residues through classical and nonclassical hydrogen bonds, hydrophobic (π–σ) bonds, and electrostatic charges because of their partial hydrophilicity [30].

The study of the fluorene biodegradation pathway reveals that bacterial consortia are involved in the complete aromatic ring cleavage. Additionally, the primary degradation of fluorene is not safe until its metabolites are also completely degraded. Further research is required to explore a novel species that degrades fluorene to nontoxic end products.

## 5. Conclusions

This research targets the upper steps of the fluorene biodegradation pathway to characterize each metabolic substrate and enzyme through in silico approaches. Fluorene is a widespread, commonly used polycyclic aromatic hydrocarbon. The key metabolites of the fluorene biodegradation are 9-Fluoronol, 9-Fluorenone, 1,1a-Dihydroxy-1-hydrofluoren-9-one, 2,3-Dihydroxy-2′-carboxybiphenyl, and 2-Hydroxy-6-oxo-6-(2-carboxyphenyl)-hexa-2,4-dienoate. They have several mild toxicities to health and the environment, and the endpoints can cause chronic diseases like cancer, respiratory disorders, and kidney failure. Laboratory research determines fluorene biodegradation rates, including the enzymes and intermediate metabolites involved. Here, we conducted a comprehensive study that includes binding interactions between the ligand and amino acid residues at every step of the pathway, ranging from −9.4 to −6.1 kcal/mol. The result supports the effective enzymatic degradation of fluorene and its metabolites; nevertheless, it is not enough to be confirmed until it is proven in a laboratory. Computational prediction does not ensure the structure and flexibility of proteins and the toxicities of ligands without experimental validation. Hopefully, the current study will help to conduct potential in vitro research on PAH biodegradation. Moreover, molecular simulation, mutational analysis, and enzyme kinetics will be explored in further studies.

## Figures and Tables

**Table 1:** SWISS-MODEL Template criteria for homology protein modelling.

NCBI Protein ID (Ref. Sequence)	Swiss Model Template	Coverage	GMQE	Identity	Method
APV43288.1 (WP_076031629.1)	2hmj.1.A	100%	98%	99.33%	X-ray, 1.5 Å
QSR30260.1 (WP_032491529.1)	Q93UV4.1.A	100%	91%	100.00%	AlphaFold v2
ALS21084.1 (WP_062407243.1)	A0A0U2UCZ7.1.A	100%	95%	100.00%	AlphaFold v2
WGJ83788.1 (WP_101841495.1)	Q93UV4.1.A	100%	93%	99.14%	AlphaFold v2
WGJ83793.1 (WP_006897066.1)	A0A6P0EVH8.1.A	100%	93%	87.93%	AlphaFold v2
BAC75995.1 (WP_032491532.1)	A0A7D7ZE28.1.A	100%	91%	98.78%	AlphaFold v2

**Table 2:** The grid box parameters of molecular dockings.

Protein	Ligand	Grid Box Size			Grid Box Size Centre		
		x	y	z	x	y	z
Naphthalene 1,2-dioxygenase	Fluorene	68.237	71.198	56.628	−26.083	72.327	87.213
Fluoren-9-ol dehydrogenase	9-Fluoronol	69.785	55.186	58.587	−2.725	4.547	−4.055
Dibenzofuran dioxygenase	9-Fluorenone	61.745	58.489	74.339	−0.566	−0.582	−1.324
1,1a-dihydroxy-1-hydro-9-fluorenone dehydrogenase	1,1a-Dihydroxy-1-hydrofluoren-9-one	69.768	55.183	58.587	−3.082	4.60	−4.232
2′-carboxy-2,3-dihydroxybiphenyl 1,2-dioxygenase	2,3-Dihydroxy-2′-carboxybiphenyl	51.377	48.468	51.660	−0.0016	−1.271	0.494
2-hydroxy-6-oxo-6-(2′-carboxyphenyl)-hexa-2,4-dienoate hydrolase	2-Hydroxy-6-oxo-6-(2-carboxyphenyl)-hexa-2,4-dienoate	103.298	61.190	63.578	−3.140	−1.038	−0.283

**Table 3:** Primary information and structures of ligands (substrates).

Ligand (Substrate) with Molecular Formula	Structure	SMILES ID	M. Weight (g/mol)
Fluorene (C_13_H_10_)	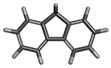	C1C2=CC=CC=C2C3=CC=CC=C31	166.22
9-Fluorenol (C_13_H_10_O)	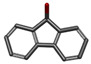	C1=CC=C2C(=C1)C(C3=CC=CC=C32)O	182.22
9-Fluorenone (C_13_H_8_O)	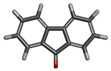	C1=CC=C2C(=C1)C3=CC=CC=C3C2=O	180.20
1,1a-Dihydroxy-1-hydrofluoren-9-one (C_13_H_10_O_3_)	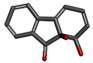	C1=CC=C2C(=C1)C3=CC=C[C@H]([C@@]3(C2=O)O)O	214.22
2,3-Dihydroxy-2′-carboxybiphenyl (C_13_H_10_O_4_)	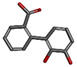	C1=CC=C(C(=C1)C2=C(C(=CC=C2)O)O)C(=O)O	230.22
2-Hydroxy-6-oxo-6-(2-carboxyphenyl)-hexa-2,4-dienoate (C_13_H_8_O_6_^−2^)	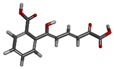	C1=CC=C(C(=C1)C(=CC=CC(=O)C(=O)O)[O-])C(=O)[O-]	260.20

**Table 4:** Toxicity classes and probability levels of ligands.

Ligand	Toxicity Class	LD50 (mg/kg)	Organ Toxicity Probability	Toxicity Endpoints Probability
Fluorene	4	620	Neurotoxicity (0.76)	Carcinogenicity (0.81), Mutagenicity (0.79), Blood–Brain Barrier (BBB) (0.97), Ecotoxicity (0.84)
9-Fluoronol	5	5000	Neurotoxicity (0.52)	Mutagenicity (0.72), BBB (0.82), Ecotoxicity (0.69)
9-Fluorenone	4	1070	Neurotoxicity (0.70)	Carcinogenicity (0.53), BBB (0.89), Ecotoxicity (0.78)
1,1a-Dihydroxy-1-hydrofluoren-9-one	4	487	Respiratory toxicity (0.67)	Carcinogenicity (0.68), Mutagenicity (0.50), BBB (0.61)
2,3-Dihydroxy-2′-carboxybiphenyl	4	800	Respiratory toxicity (0.61), Nephrotoxicity (0.66)	Carcinogenicity (0.65), BBB (0.61)
2-Hydroxy-6-oxo-6-(2-carboxyphenyl)-hexa-2,4-dienoate	4	500	Nephrotoxicity (0.64)	BBB (0.80)

**Table 5:** Identical proteomic information on selected enzymes and their sources.

Enzyme Name	Gene (Entry)	NCBI Protein ID (Ref. Sequence)	Amino Acids	Location	Species	Group
Naphthalene 1,2-dioxygenase	*ndoB* (K14579)	APV43288.1 (WP_076031629.1)	449	Plasmid	*Pseudomonas* sp.	Gram-Negative
Fluoren-9-ol dehydrogenase	*flnB* (R05349)	QSR30260.1 (WP_032491529.1)	357	Chromosome	*Nocardioides* sp.	Gram-Positive
Dibenzofuran dioxygenase	*dbfA1* (K14599)	ALS21084.1 (WP_062407243.1)	431	Chromosome	*Paenibacillus* sp.	Gram-Positive
1,1a-dihydroxy-1-hydro-9-fluorenone dehydrogenase	*flnB* (K14601)	WGJ83788.1 (WP_101841495.1)	349	Chromosome	*Gordonia* sp.	Gram-Positive
2′-carboxy-2,3-dihydroxybiphenyl 1,2-dioxygenase	*flnD1* (K14602)	WGJ83793.1 (WP_006897066.1)	290	Chromosome	*Gordonia* sp.	Gram-Positive
2-hydroxy-6-oxo-6-(2′-carboxyphenyl)-hexa-2,4-dienoate hydrolase	*flnE* (K14604)	BAC75995.1 (WP_032491532.1)	328	Plasmid	*Terrabacter* sp.	Gram-Positive

**Table 6:** Physicochemical properties of selected proteins.

NCBI Protein ID	Molecular Formula	Molecular Weight	pI	Instability Index	Aliphatic Index	GRAVY
WP_076031629.1	C_2214_H_3355_N_603_O_668_S_15_	49,588.53	5.77	34.11	72.76	−0.371
WP_032491529.1	C_1696_H_2611_N_479_O_526_S_12_	38,512.00	4.69	32.40	80.45	−0.155
WP_062407243.1	C_2266_H_3377_N_597_O_646_S_14_	49,767.19	5.88	31.80	73.32	−0.514
WP_101841495.1	C_1669_H_2567_N_471_O_514_S_12_	37,839.31	4.72	30.44	80.63	−0.165
WP_006897066.1	C_1366_H_2140_N_378_O_408_S_10_	30,706.91	4.79	38.25	93.28	0.124
WP_032491532.1	C_1563_H_2452_N_464_O_474_S_6_	35,519.85	5.89	28.84	80.34	−0.256

**Table 7:** Swiss-model templates of predicted 3D structures and active site scores of the proteins.

NCBI Protein ID	Swissmodel Template	No. of Binding Pockets	Highest Score	Probability	No. of Residues	Average Conservation
WP_076031629.1	2hmj.1.A	09	23.14	0.870	17	1.052
WP_032491529.1	Q93UV4.1.A	04	73.88	0.991	49	1.456
WP_062407243.1	A0A0U2UCZ7.1.A	08	16.82	0.787	19	1.032
WP_101841495.1	Q93UV4.1.A	04	74.36	0.991	49	1.474
WP_006897066.1	A0A6P0EVH8.1.A	04	27.29	0.901	20	1.446
WP_032491532.1	A0A7D7ZE28.1.A	03	13.67	0.711	23	0.572

**Table 8:** Protein 3D structure validation scores.

NCBI Protein ID	ERRAT		PROCHECK (Ramachandran Plot)				3D Verification
	Factor	Chain	Most Allowed Region	Additional Allowed Region	Generously Allowed Region	Disallowed Region	≥0.1 3D/1D Profile
WP_076031629.1	94.279	E	90.0%	9.7%	0.0%	0.3%	97.53%
WP_032491529.1	99.403	A	92.8%	6.9%	0.3%	0.0%	90.76%
WP_062407243.1	94.787	A	91.9%	7.5%	0.0%	0.5%	94.43%
WP_101841495.1	99.392	A	93.3%	6.3%	0.3%	0.0%	90.54%
WP_006897066.1	94.643	A	90.5%	9.1%	0.0%	0.4%	81.38%
WP_032491532.1	97.297	A	86.2%	11.6%	1.4%	0.7%	96.95%

**Table 9:** Molecular docking analysis of fluorene biodegradation pathway.

Protein	Ligand	PrankWeb (kcal/mol)	PyRx (v0.8) (kcal/mol)	rmsd/lb (Å)	rmsd/ub (Å)
Naphthalene 1,2-dioxygenase	Fluorene	−9.418	−9.4	00	00
Fluoren-9-ol dehydrogenase	9-Fluoronol	−7.640	−7.7	00	00
Dibenzofuran dioxygenase	9-Fluorenone	−6.617	−6.6	00	00
1,1a-dihydroxy-1-hydro-9-fluorenone dehydrogenase	1,1a-Dihydroxy-1-hydrofluoren-9-one	−8.353	−8.5	00	00
2′-carboxy-2,3-dihydroxybiphenyl 1,2-dioxygenase	2,3-Dihydroxy-2′-carboxybiphenyl	−6.452	−6.2	00	00
2-hydroxy-6-oxo-6-(2′-carboxyphenyl)-hexa-2,4-dienoate hydrolase	2-Hydroxy-6-oxo-6-(2-carboxyphenyl)-hexa-2,4-dienoate	−7.351	−6.1	00	00

**Table 10:** The binding interactions of ligands with amino acid residues.

Ligand	Residues	Distance (Å)	Category	Type
Fluorene	HIS E:208	4.86	Electrostatic	Pi-Charge
	PHE E:202	4.76	Hydrophobic	Pi Hydrophobic
	LEU E:307	3.63	Hydrophobic	Mixed Pi/Alkyl Hydrophobic (π–σ)
	VAL E:209	4.83	Hydrophobic	Mixed Pi/Alkyl Hydrophobic (π–σ)
9-Fluoronol	ARG A:190	4.06, 4.33	Electrostatic	Pi-Charge
	GLU A:195	3.96	Electrostatic	Pi-Charge
	PHE A:251	5.26	Hydrophobic	Pi Hydrophobic
	HIS A:230	2.77	Hydrogen Bond	Nonclassical (C–H Bond)
	GLY A:229	3.69	Hydrogen Bond	Nonclassical (C–H Bond)
	SER A:175	2.14	Hydrogen Bond	Classical (Conventional H-Bond)
9-Fluorenone	ASP A:205	3.17, 3.63	Electrostatic	Pi-Charge
	ASN A:202	2.84	Hydrogen Bond	Nonclassical (C–H Bond)
	GLN A:412	2.22	Hydrogen Bond	Classical (Conventional H-Bond)
1,1a-Dihydroxy-1-hydrofluoren-9-one	ARG A:182	3.51	Electrostatic	Pi-Charge
	PHE A:243	3.83	Hydrophobic	Pi Hydrophobic
	ARG A:182	2.79	Hydrogen Bond	Classical (Conventional H-Bond)
	TRP A:184	1.89, 2.67	Hydrogen Bond	Classical (Conventional H-Bond)
2,3-Dihydroxy-2′-carboxybiphenyl	ARG A:163	4.53	Hydrophobic	Mixed Pi/Alkyl Hydrophobic (π–σ)
	ARG A:246	3.83, 5.19	Hydrophobic	Mixed Pi/Alkyl Hydrophobic (π–σ)
	GLN A:247	1.92	Hydrogen Bond	Classical (Conventional H-Bond)
2-Hydroxy-6-oxo-6-(2 carboxyphenyl)-hexa-2,4-dienoate	PHE A:270	3.49	Hydrophobic	Mixed Pi/Alkyl Hydrophobic (π–σ)
	PHE A:270	2.31	Hydrogen Bond	Classical (Conventional H-Bond)
	ALA A:268	2.46	Hydrogen Bond	Classical (Conventional H-Bond)
	ILE A:265	1.92	Hydrogen Bond	Classical (Conventional H-Bond)
	ARG A:263	2.47	Hydrogen Bond	Classical (Conventional H-Bond)
	SER A:262	2.78, 2.93, 3.08	Hydrogen Bond	Classical (Conventional H-Bond)

## Data Availability

The original contributions presented in this study are included in the article. Further queries can be directed to the corresponding author.
